# Do host genetic traits in the bacterial sensing system play a role in the development of *Chlamydia trachomatis*-associated tubal pathology in subfertile women?

**DOI:** 10.1186/1471-2334-6-122

**Published:** 2006-07-21

**Authors:** Janneke E den Hartog, Sander Ouburg, Jolande A Land, Joseph M Lyons, James I Ito, A Salvador Peña, Servaas A Morré

**Affiliations:** 1 Research Institute Growth and Development (GROW) and Department of Obstetrics and Gynaecology, Academic Hospital Maastricht, P.O. Box 5800, 6202 AZ Maastricht, The Netherlands; 2 Laboratory of Immunogenetics, Section Immunogenetics of Infectious Diseases, Department of Pathology, VU University Medical Center, Van der Boechorststraat 7, 1081 BT Amsterdam, The Netherlands; 3 Department of Infectious Diseases, City of Hope National Medical Center and Beckman Research Institute, 1500 E. Duarte Road, Duarte, California 91010, USA; 4 Department of Internal Medicine, Section Infectious Diseases, VU University Medical Center, Van der Boechorststraat 7, 1081 BT Amsterdam, The Netherlands; 5 Department of Medical Microbiology, Academic Hospital Maastricht, P.O. Box 5800, 6202 AZ Maastricht, The Netherlands; 6 On behalf of the ICTI consortium (Integrated approach to the study of *Chlamydia trachomatis *Infections) and the EpiGenChlamydia consortium

## Abstract

**Background:**

In women, *Chlamydia (C.) trachomatis* upper genital tract infection can cause distal tubal damage and occlusion, increasing the risk of tubal factor subfertility and ectopic pregnancy. Variations, like single nucleotide polymorphisms (SNPs), in immunologically important host genes are assumed to play a role in the course and outcome of a *C. trachomatis* infection. We studied whether genetic traits (carrying multiple SNPs in different genes) in the bacterial sensing system are associated with an aberrant immune response and subsequently with tubal pathology following a *C. trachomatis* infection. The genes studied all encode for pattern recognition receptors (PRRs) involved in sensing bacterial components.

**Methods:**

Of 227 subfertile women, serum was available for *C. trachomatis* IgG antibody testing and genotyping (common versus rare allele) of the PRR genes TLR9, TLR4, CD14 and CARD15/NOD2. In all women, a laparoscopy was performed to assess the grade of tubal pathology. Tubal pathology was defined as extensive peri-adnexal adhesions and/or distal occlusion of at least one tube.

**Results:**

Following a *C. trachomatis* infection (i.e. *C. trachomatis* IgG positive), subfertile women carrying two or more SNPs in *C. trachomatis* PRR genes were at increased risk of tubal pathology compared to women carrying less than two SNPs (73% vs 33% risk). The differences were not statistically significant (P = 0.15), but a trend was observed.

**Conclusion:**

Carrying multiple SNPs in *C. trachomatis* PRR genes tends to result in an aberrant immune response and a higher risk of tubal pathology following a *C. trachomatis* infection. Larger studies are needed to confirm our preliminary findings.

## Background

A large variation exists in the individual response to a *Chlamydia *(*C.*) *trachomatis *infection. Some women clear a *C. trachomatis *infection adequately without developing tissue damage, whereas others get a persistent infection which may ascend to the upper genital tract, increasing the risk of tubal damage and subfertility. The susceptibility, course and outcome of infectious diseases are determined by environmental factors, virulence factors of the pathogen and host factors.

Immunogenetic studies evaluate the role of genetic variations in immunologically important host genes as determinants of the susceptibility, course and outcome of infectious diseases. Among these variations are single nucleotide polymorphisms (SNPs), in which one nucleotide has been substituted, inserted or deleted. This may lead to synthesis of a potentially aberrant protein, or to up- or downregulation of the normal protein, and subsequently to an aberrant immune response, increasing the risk of late sequelae of infectious diseases (e.g. tubal pathology following a *C. trachomatis *infection).

In the present study, we have evaluated SNPs in genes encoding for pattern recognition receptors (PRRs). PRRs are present on or in circulating cells of the innate immune system (e.g. macrophages) and local cells (e.g. epithelial cells of the upper genital tract). PRRs are involved in the bacterial sensing pathways of the innate immune system by recognizing the so-called pathogen-associated molecular patterns (PAMPs), which are pathogen-specific cell wall components or intracellular components. Since different PRRs recognize different PAMPs, pathogen recognition and initiation of the immune response is a complex and flexible system.

Carrying a SNP in a single PRR may not result in a large effect on disease severity, since other PRRs may compensate for the partial loss of function in a specific pathogen recognition route. Subsequently, SNPs in only one PRR may not play a significant role as risk factors for the development of *C. trachomati*s-associated tubal pathology, as shown for the PRR toll-like receptor (TLR) 4 [1] and its co-receptor cluster of differentiation (CD) 14 [2]. However, carrying multiple SNPs in one gene or in multiple genes (in so-called carrier traits) may be associated with an increased risk of tubal pathology. Smirnova *et al*. (2003) [3] have found that combinations of TLR4 variants are markedly more common in patients with meningococcal infections, whereas single variants are not over-represented in those patients. In studies on gastrointestinal malignancies, it has been concluded that carrying multiple pro-inflammatory polymorphisms is associated with an increased risk of gastric cancer [4,5]. Furthermore, studies on the relationship between caspase recruitment domain (CARD) 15/nucleotide oligomerisation domain (NOD) 2 genetic variants, of which SNP8, SNP12 and SNP13 are most studied, and Crohn's disease have shown that compound heterozygous subjects (carriers of two different genetic variants, e.g. SNP12 genotype 1.2 and SNP13 genotype 1.2) have a higher risk of Crohn's disease as compared to homozygous subjects (carriers of the same genetic variant on both chromosomes, e.g. SNP12 genotype 2.2) [6,7].

Analogous to these findings, we hypothesized that carrying multiple genetic variations in multiple PRRs (in a so-called carrier trait) may increase the risk of *C. trachomatis*-associated tubal pathology in subfertile women. According to their biological function (recognition of *C. trachomatis *PAMPs: see Table 1), four PRRs were selected: TLR9, TLR4, CD14 and CARD15/NOD2. Five relatively common SNPs, which are assumed to influence the receptor function, in these four PRR genes were studied in this carrier trait analysis (see Table 1).

**Table 1:** The pattern recognition receptors (PRRs), which recognize *C. trachomatis *pathogen-associated molecular patterns (PAMPs), and the single nucleotide polymorphisms (SNPs) studied

PRR	PAMP	SNP
TLR9	CpG-rich motifs	-1237 T>C and +2848 G>A
TLR4	LPS and HSP	+896 A>G
CD14	LPS and HSP (co-receptor of TLR4)	-260 C>T
CARD15/NOD2	Peptidoglycans	Leu1007fsinsC (SNP13)

TLR = toll-like receptor; CD = cluster of differentiation; CARD = caspase recruitment domain; NOD = nucleotide oligomerisation domain; CpG = cytosine-phosphate-guanine; LPS = lipopolysaccharide; HSP = heat shock protein; T = thymine; C = cytosine; G = guanine; A = adenine; Leu = leucine-rich repeat domain; fsins = frameshift insertion.

## Methods

### Study population

The study was performed in women who visited the Academic Hospital Maastricht between December 1990 and November 2000 because of subfertility. In all patients blood was drawn at their initial visit for a Chlamydia IgG antibody test (CAT). All spare sera were cryopreserved. Only patients who had undergone a laparoscopy and tubal testing as part of their fertility work-up were included in the present study. Since the prevalence of SNPs may depend on ethnical background, only Dutch Caucasian women were included. Patients who had undergone previous pelvic surgery (except for an uneventful appendectomy or Caesarean section) were excluded. In the Netherlands, for retrospective analysis of anonymized patient data and stored sera no ethical committee approval is required. In the fertility clinic of the Academic Hospital Maastricht, all couples are informed at intake about possible use of their anonymized data and stored sera for research purposes, and a "no objection procedure" is followed. Only patients having not objected participated in the present study.

Two independent investigators, who were unaware of the CAT results, scored 259 successive laparoscopy reports to assess the grade of tubal pathology. Tubal pathology was defined as extensive peri-adnexal adhesions and/or distal occlusion of at least one tube [8]. In case of disagreement, consensus was reached by consultation.

Of the 259 women who underwent a laparoscopy, 43 (17%) had tubal pathology (according to the above-mentioned definition) and 184 (71%) had no tubal pathology (no peri-adnexal adhesions and patent tubes), and these 227 women participated in the present study. Thirty-two women (12%) had minor or non-*C. trachomatis*-related abnormalities (any peri-adnexal adhesions and/or proximal occlusion of at least one tube) and were excluded.

### *C. trachomatis *IgG antibody testing

IgG antibodies to *C. trachomatis *were detected using the species-specific *Chlamydia pneumoniae *IgG micro-immunofluorescence (MIF) test (AniLabsystems, Finland), as described previously [9]. This species-specific test is able to detect IgG antibodies to both *C. pneumoniae *and *C. trachomatis *(using an antigen derived from a *C. trachomatis *LGV strain, serovar L2). We have previously studied the test performances of five commercially available *C. trachomatis *IgG tests, including the *C. trachomatis *IgG spot in the *C. pneumoniae *MIF (AniLabsystems) [10]. In our hands, the *C. trachomatis *IgG titre obtained by the *C. pneumoniae *MIF (AniLabsystems) had the best predictive value for tubal factor subfertility [10]. Therefore, we have used this test in the present study. The cut-off titre used for a positive test was 32.

### Immunogenetic analysis

For the immunogenetic analyses, genomic DNA was extracted from the cryopreserved serum samples using either the MagNaPure LC isolator according to the manufacturers' instructions (Roche Molecular Biochemicals, Germany) or the High Pure PCR Template Preparation (HPPTP) Kit according to the manufacturers' instructions (Roche Molecular Biochemicals, Germany). Both techniques provide enough DNA for reproducible genetic analyses. Genotyping was performed using a polymerase chain reaction (PCR)-based restriction fragment length polymorphism (RFLP)-assay or TaqMan-assay as described previously [2,11-13]. The SNPs studied are summarized in Table 1. Chromosomal locations and further information on the genes studied are: TLR9 chromosomal location 3p21.3, TLR9 -1237 T>C rs5743836 and TLR9 +2848 G>A rs352140; TLR4 chromosomal location 9q32-q33, TLR4 +896 A>G rs4986790; CD14 chromosomal location 5q31.1, CD14 -260 C>T rs25691909; CARD15/NOD2 chromosomal location 16q21, CARD15/NOD2 Leu1007fsinsC (SNP13) rs2066847. For ethnically-matched background genotyping, genomic DNA was extracted from whole blood of 97 healthy Dutch Caucasian employees of the VU University Medical Center. They gave written informed consent for use of their anonymized sera to serve as control sera for genetic research purposes.

### Statistical methods

The genotype distribution was tested for Hardy-Weinberg equilibrium to assess Mendelian inheritance. Fisher's exact or χ^2 ^tests were used to compare the single genotypes between *C. trachomatis *IgG-positive and IgG-negative subfertile women with and without tubal pathology and the healthy control group. Subsequently, the single genotypes were used to define carrier traits. The carrier traits were tested in χ^2 ^and trend analyses. *P *< 0.05 was considered statistically significant.

## Results

Of all 227 subfertile women participating in the present study, 43 (19%) had tubal pathology, whereas 184 (81%) did not have tubal pathology. *C. trachomatis *IgG antibodies were present in 39 women, of whom 26 (67%) had tubal pathology and 13 (33%) did not have tubal pathology. *C. trachomatis *IgG antibodies were absent in 188 women, of whom 17 (9%) had tubal pathology and 171 (91%) did not have tubal pathology.

For all genes studied, the genotype distribution was in Hardy-Weinberg equilibrium in the subfertile women and the ethnically-matched control group.

The genotype distribution did not differ between subfertile women, the *C. trachomatis *IgG-positive subgroup of subfertile women and the healthy control group (Table 2), indicating that the subfertile women participating in the study reflect an average Dutch Caucasian population regarding the genotype distribution.

**Table 2:** The risk of tubal pathology (TP) in relation to the genotype of the single genes studied.

		1.1		1.2 and 2.2	
		
		n	Risk of TP	n	Risk of TP
TLR9 -1237 T>C	All subfertile women	155 (68%)	20%	72 (32%)	17%
	CT+ subfertile women	26 (67%)	62%	13 (33%)	77%
	Control group	66 (68%)	-	31 (32%)	-
TLR9 +2848 G>A	All subfertile women	45 (20%)	18%	182 (80%)	19%
	CT+ subfertile women	6 (15%)	50%	33 (85%)	70%
	Control group	15 (15%)	-	82 (85%)	-
TLR4 +896 A>G^a^	All subfertile women	200 (88%)	19%	27 (12%)	22%
	CT+ subfertile women	33 (85%)	64%	6 (15%)	83%
	Control group	87 (90%)	-	10 (10%)	-
CD14 -260 C>T^b^	All subfertile women	60 (26%)	17%	167 (74%)	20%
	CT+ subfertile women	12 (31%)	67%	27 (69%)	67%
	Control group	26 (27%)	-	71 (73%)	-
CARD15/NOD2	All subfertile women	211 (93%)	18%	16 (7%)	25%
Leu1007fsinsC	CT+ subfertile women	37 (95%)	65%	2 (5%)	100%
(SNP13)	Control group	95 (98%)	-	2 (2%)	-

All subfertile women: n = 227, of whom 19% has tubal pathology (TP).

CT + (*C. trachomatis *IgG-positive) subfertile women: n = 39, of whom 67% has TP.

Control group: n = 97 ethnically-matched healthy employees of the VU University Medical Center.

1.1 = normal genotype (homozygous for the common allele); 1.2 = heterozygous SNP carrier (one common allele and one rare allele); 2.2 = homozygous SNP carrier (homozygous for the rare allele).

^a ^Adapted from Morré *et al*., 2003 [1].

^b ^Adapted from Ouburg *et al*., 2005 [2].

### Single gene analysis

**Figure 1:**
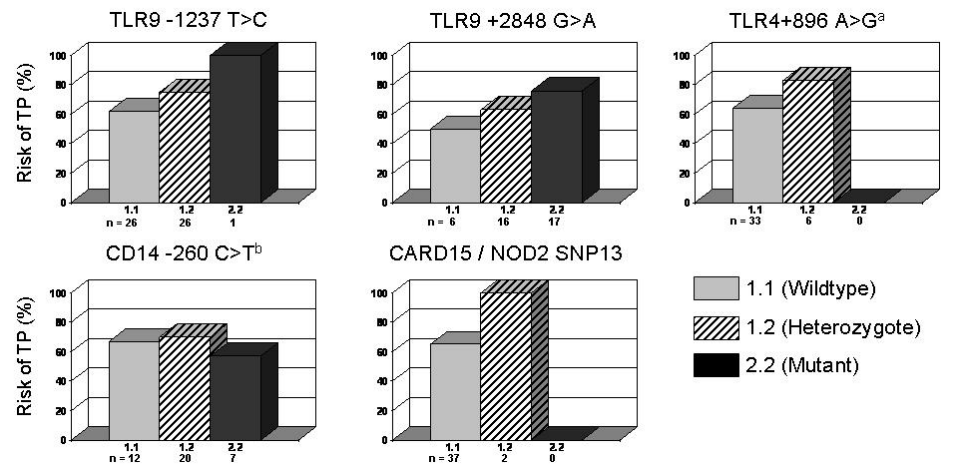
The risk of tubal pathology (TP) in *C. trachomatis *IgG-positive subfertile women in relation to the genotype of the single pattern recognition receptor genes. ^a ^Adapted from Morré *et al*., 2003 [1]. ^b ^Adapted from Ouburg *et al*., 2005 [2].

The risk of tubal pathology was assessed in all subfertile women and in the *C. trachomatis *IgG-positive subgroup in relation to the genotype of TLR9, TLR4, CD14 and CARD15/NOD2 (Table 2; Figure 1). An increasing risk of tubal pathology was observed across the genotypes in all genes except CD14. Carrying SNPs in these genes increased the risk of tubal pathology (on average almost 20%). These differences did not reach statistical significance. These single genotypes were used to define carrier traits.

### Carrier trait analysis

**Figure 2:**
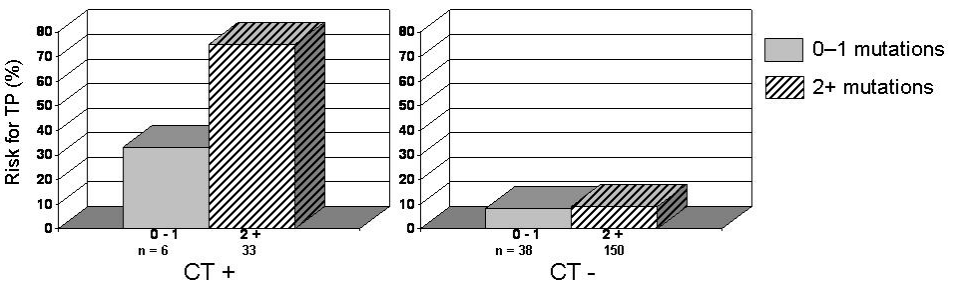
The risk of tubal pathology (TP) in *C. trachomatis *IgG-positive (CT+) and IgG-negative (CT-) subfertile women in relation to carrying five single nucleotide polymorphisms (SNPs) in four pattern recognition receptor genes.

The SNPs in the single genes were combined in carrier traits. The risk of tubal pathology was assessed in *C. trachomatis *IgG-positive and IgG-negative subfertile women in relation to the number of SNPs. Carrying two or more SNPs did not influence the risk of tubal pathology in *C. trachomatis *IgG-negative women as compared to *C. trachomatis *IgG-negative women carrying less than two SNPs (9% vs. 8% risk respectively; Figure 2). However, carrying multiple SNPs doubled the risk of tubal pathology in *C. trachomatis *IgG-positive women as compared to *C. trachomatis *IgG-positive women with less than two SNPs (73% vs. 33% risk respectively; Figure 2). These differences did not reach statistical significance (*P *= 0.15) but a clear trend was observed.

## Discussion

Over the last decade, immunogenetic studies have provided insight in the pathogenesis of and susceptibility to infectious diseases. So far, the role of SNPs in immunologically relevant genes has been established in numerous diseases, e.g. sexually transmitted infections [14-16] and inflammatory bowel diseases (Crohn's disease and ulcerative colitis) [17,18]. In this study, we analyzed the role of single SNPs and multiple SNPs in multiple genes (in a so-called carrier trait) as risk factors of *C. trachomatis*-related tubal pathology and we confirmed our hypothesis that a carrier trait based on genes in bacterial sensing pathways had a stronger association with the risk of tubal pathology than a single gene analysis.

Recent studies have shown the value of genetic traits in complex diseases. Carrying multiple SNPs in the same gene, or multiple SNPs in multiple genes, has been associated with an increased risk of infectious diseases and malignancies [3,4,6,7]. Analogous to these findings, we hypothesized that the disregulation of the immune response by the presence of multiple SNPs may lead to an even higher risk of tubal pathology following a *C. trachomatis *infection as compared to carrying a single SNP.

In this study, we investigated the role of five SNPs in four genes which are assumed to play a role in the recognition of *C. trachomatis *(see Table 1). An adequate recognition of *C. trachomatis *by PRRs is the first step in the immune response. Recent studies have shown that TLR1-9 are expressed in the human female genital tract. TLR4 and its co-receptor CD14 are predominantly expressed in the fallopian tubes, where they may play an important role in the innate host defence mechanism against ascending *C. trachomatis *infections [19-21]. Regarding CARD15/NOD2, it is not clear whether it plays a role in the *C. trachomatis *recognition in the genital tract, although NOD proteins have been shown to be involved in the intracellular sensing of *C. pneumoniae *in endothelial cells [22].

Our data show a doubling of the risk of tubal pathology in *C. trachomatis *IgG-positive women carrying two or more SNPs as compared to *C. trachomatis *IgG-positive women carrying less than two SNPs (73% vs. 33% risk). The differences did not reach statistical significance (*P *= 0.15) due to the small sample size (227 subfertile women in total, including 39 *C. trachomatis *IgG-positive subfertile women, of whom 67% has tubal pathology). If the association found in this pilot study would be confirmed in a larger cohort, a 50% increase in sample size (to 341 subfertile women) would result in a significant difference (*P *= 0.047) between *C. trachomatis *IgG-positive subfertile women carrying two or more SNPs versus *C. trachomatis *IgG-positive subfertile women carrying less than two SNPs regarding the risk of tubal pathology. A 100% increase in sample size (to 454 subfertile women) would result in a strong association (*P *= 0.016; OR: 5.3) between carrying two or more SNPs and an increased risk of tubal pathology. Increasing the sample size twofold would not be possible however in our setting in a reasonable time frame. Although the present study was performed in a large fertility clinic and patients were included during a ten-year period, the number of affected women remained small, due to the low prevalence of IgG-positivity in combination with both carrying multiple SNPs and tubal pathology.

From our results it can be concluded that an adequate recognition of the pathogen at the site of infection seems to be a relevant step in the immune response, and that carrying multiple SNPs in multiple *C. trachomatis *PRR genes tends to increase the risk of an aberrant immune response and tubal pathology. To draw significant conclusions, our hypothesis should be retested in further studies using a larger cohort.

As expected, a difference in risk of tubal pathology between *C. trachomatis *IgG-positive women and *C. trachomatis *IgG-negative women was observed. In previous studies, the presence of *C. trachomatis *IgG antibodies, an indicator of a previous *C. trachomatis *infection, has been associated with tubal pathology [23]. Our data show that carrying multiple SNPs in bacterial sensing pathways and a previous *C. trachomatis *infection synergistically enhance the risk of tubal pathology, while carrying these SNPs does not influence the risk of tubal pathology in the absence of a previous *C. trachomatis *infection (Figure 2).

## Conclusion

We hypothesized that carrier traits (i.e. carrying multiple SNPs in multiple genes) that likely result in an aberrant immune response are associated with tubal pathology following a *C. trachomatis *infection. In 227 subfertile women, we studied five variations in four genes encoding for pattern recognition receptors, which recognize several pathogen-associated molecular patterns of *C. trachomatis*. The presence of two or more SNPs tends to correlate with an increased risk of tubal pathology following a *C. trachomatis *infection as compared to a lower number of SNPs. Further studies in a larger cohort are needed to confirm our preliminary findings. An adequate recognition of *C. trachomatis *by receptors in the genital tract seems to be a relevant step in the immune response, and may play a role in protecting the host against the development of late sequelae following a *C. trachomatis *infection.

## Competing interests

The author(s) declare that they have no competing interests.

## Authors' contributions

JEdH: Data acquisition, statistical analysis, drafting the manuscript.

SO: Data acquisition, statistical analysis, critically revising for immunogenetic content.

JAL: Sample collection, critically revising for medical content.

JML: Partial gene selection based on knock out work, critically reading the manuscript.

JII: Partial gene selection based on knock out work, critically reading the manuscript. 

ASP: Critically revising for immunogenetic content.

SAM: Study design, conception and coordination, statistical analysis, critically revising for immunogenetic content.

*All authors contributed to writing of the final manuscript*.

*All authors read and approved the final manuscript*.

## Pre-publication history

The pre-publication history for this paper can be accessed here:



## References

[B1] Morré SA, Murillo LS, Bruggeman CA, Peña AS (2003). The role that the functional Asp299Gly polymorphism in the toll-like receptor-4 gene plays in the susceptibility to *Chlamydia trachomatis*-associated tubal infertility. J Infect Dis.

[B2] Ouburg S, Spaargaren J, Den Hartog JE, Land JA, Fennema JSA, Pleijster J, Peña AS, Morré SA (2005). The *CD14 *functional gene polymorphism -260 C>T is not involved in either the susceptibility to *Chlamydia trachomatis *infection or the development of tubal pathology. BMC Infect Dis.

[B3] Smirnova I, Mann N, Dols A, Derkx HH, Hibberd ML, Levin M, Beutler B (2003). Assay of locus-specific genetic load implicates rare Toll-like receptor 4 mutations in meningococcal susceptibility. Proc Natl Acad Sci USA.

[B4] El-Omar EM, Rabkin CS, Gammon MD, Vaughan TL, Risch HA, Schoenberg JB, Stanford JL, Mayne ST, Goedert J, Blot WJ (2003). Increased risk of noncardia gastric cancer associated with proinflammatory cytokine gene polymorphisms. Gastroenterology.

[B5] Machado JC, Figueiredo C, Canedo P, Pharoah P, Carvalho R, Nabais S, Castro Alves C, Campos ML, Van Doorn LJ, Caldos C (2003). A proinflammatory genetic profile increases the risk for chronic atrophic gastritis and gastric carcinoma. Gastroenterology.

[B6] Hugot JP, Chamaillard M, Zouali H, Lesage S, Cézard JP, Belaiche J, Almer S, Tysk C, O'Morain CA, Gassull M (2001). Association of NOD2 leucine-rich repeat variants with susceptibility to Crohn's disease. Nature.

[B7] Heresbach D, Gicquel-Douabin V, Birebent B, D'Halluin PN, Heresbach-Le Berre N, Dreano S, Siproudhis L, Dabadie A, Gosselin M, Mosser J (2004). NOD2/CARD15 gene polymorphisms in Crohn's disease: a genotype-phenotype analysis. Eur J Gastroenterol Hepatol.

[B8] Land JA, Evers JLH, Goossens VJ (1998). How to use Chlamydia antibody testing in subfertility patients. Hum Reprod.

[B9] Den Hartog JE, Land JA, Stassen FRM, Slobbe-Van Drunen MEP, Kessels AGH, Bruggeman CA (2004). The role of chlamydia genus-specific and species-specific IgG-antibody testing in predicting tubal disease in subfertile women. Hum Reprod.

[B10] Land JA, Gijsen AP, Kessels AGH, Slobbe MEP, Bruggeman CA (2003). Performance of five serological chlamydia antibody tests in subfertile women. Hum Reprod.

[B11] Morré SA, Murillo LS, Spaargaren J, Fennema JSA, Peña AS (2002). Role of the toll-like receptor 4 Asp299Gly polymorphism in susceptibility to *Candida albicans *infection. J Infect Dis.

[B12] Murillo LS, Crusius JBA, Van Bodegraven AA, Alizadeh BZ, Peña AS (2002). *CARD15 *gene and the classification of Crohn's disease. Immunogenetics.

[B13] Lammers KM, Ouburg S, Morré SA, Crusius JBA, Gionchetti P, Rizzello F, Morselli C, Caramelli E, Conte R, Poggioli G (2005). Combined carriership of *TLR9 *-1237C and *CD14 *-260T alleles enhances the risk of developing chronic relapsing pouchitis. World J Gastroenterol.

[B14] Jeremias J, Giraldo P, Durrant S, Ribeiro-Filho A, Witkin SS (1999). Relationship between *Ureaplasma urealyticum *vaginal colonization and polymorphism in the interleukin-1 receptor antagonist gene. J Infect Dis.

[B15] Kinnunen AH, Surcel HM, Lehtinen M, Karhukorpi J, Tiitinen A, Halttunen M, Bloigu A, Morrison RP, Karttunen R, Paavonen J (2002). HLA DQ alleles and interleukin-10 polymorphism associated with *Chlamydia trachomatis*-related tubal factor infertility: a case-control study. Hum Reprod.

[B16] Wang C, Tang J, Geisler WM, Crowley-Nowick PA, Wilson CM, Kaslow RA (2005). Human leukocyte antigen and cytokine gene variants as predictors of recurrent *Chlamydia trachomatis *infection in high-risk adolescents. J Infect Dis.

[B17] Franchimont D, Vermeire S, El Housni H, Pierik M, Van Steen K, Gustot T, Quertinmont E, Abramowicz M, Van Gossum A, Devière J (2004). Deficient host-bacteria interactions in inflammatory bowel disease? The toll-like receptor (TLR)-4 Asp299gly polymorphism is associated with Crohn's disease and ulcerative colitis. Gut.

[B18] Peeters H, Vander Cruyssen B, Laukens D, Coucke P, Marichal D, Van Den Berghe M, Cuvelier C, Remaut E, Mielants H, De Keyser F (2004). Radiological sacroiliitis, a hallmark of spondylitis, is linked with CARD15 gene polymorphisms in patients with Crohn's disease. Ann Rheum Dis.

[B19] Pioli PA, Amiel E, Schaefer TM, Connolly JE, Wira CR, Guyre PM (2004). Differential expression of Toll-like receptors 2 and 4 in tissues of the human female reproductive tract. Infect Immun.

[B20] Schaefer TM, Desouza K, Fahey JV, Beagley KW, Wira CR (2004). Toll-like receptor (TLR) expression and TLR-mediated cytokine/chemokine production by human uterine epithelial cells. Immunology.

[B21] Fazeli A, Bruce C, Anumba DO (2005). Characterization of Toll-like receptors in the female reproductive tract in humans. Hum Reprod.

[B22] Opitz B, Förster S, Hocke AC, Maass M, Schmeck B, Hippenstiel S, Suttorp N, Krüll M (2005). Nod1-mediated endothelial cell activation by *Chlamydophila pneumoniae*. Circ Res.

[B23] Punnonen R, Terho P, Nikkanen V, Meurman O (1979). Chlamydial serology in infertile women by immunofluorescence. Fertil Steril.

[B24] Morré SA, Spaargaren J, Ossewaarde JM, Land JA, Bax CJ, Dörr PJ, Oostvogel PM, Vanrompay D, Savelkoul PHM, Pannekoek Y (2006). Description of the ICTI consortium: an integrated approach to the study of *Chlamydia trachomatis *infection. Drugs Today.

